# The Role of Activated Cytotoxic T Cells in Etiopathogenesis of Periodontal Disease: Does It Harm or Does It Heal?

**DOI:** 10.1038/srep09262

**Published:** 2015-03-19

**Authors:** Emine Cifcibasi, Meral Ciblak, Bayram Kiran, Selim Badur, Erhan Firatli, Halim Issever, Serdar Cintan

**Affiliations:** 1 Istanbul University, Faculty of Dentistry, Dpt. of Periodontology, Istanbul, Turkey; 2 Istanbul University, Istanbul Faculty of Medicine, Dpt. of Microbiology and Clinical Microbiology, Virology and Fundamental Immunology Unit, Istanbul, Turkey; 3 Istanbul University, Istanbul Faculty of Medicine, Dpt. of Public Health, Istanbul, Turkey; 4 Department of Biology, Kastamonu University, Kastamonu, Turkey

## Abstract

The objective of this study was to determine the phenotypic profile of blood mononuclear cells, specifically CD8^+^/CD28^+^ cells, in patients with generalized aggressive periodontitis (GAgP) and chronic periodontitis (CP) in peripheral blood and in blood obtained from periodontal defect site which might contribute to tissue damage. 13 GAgP, 11 chronic periodontitis (CP) and 5 healthy controls (H) were included in the study. Plaque index (PI), bleeding on probing (BoP), periodontal probing depth (PPD) and clinical attachment level (CAL) were recorded. Blood from the base of periodontal defect site and peripheral blood from the antecubital vein were obtained. Relative counts of CD45^+^, CD3^+^, CD4^+^, CD8^+^/CD28^+^, CD8^+^/CD28^−^, CD19^+^, CD16^+^/CD56^+^/CD3, CD3^+^/CD16^+^/CD56^+^ receptors were determined with two color flow cytometry using monoclonal antibodies. BoP, PPD and CAL were significantly higher in both periodontitis groups than healthy controls (p <0.05). Activated cytotoxic T cells, CD8^+^/CD28^+^ cells, were significantly elevated in GAgP and CP groups compared to HC both in blood obtained from defect site and blood obtained from systemic circulation (p <0.05). GAgP and CP patients have an increased levels of activated cytotoxic T cells as a result of inflammation which may cause severe tissue damage that lead to severe and rapid loss of periodontal tissues.

Periodontitis defines a group of disease which cause inflammatory destruction in periodontal tissues and vary in age of onset and rate of progression^1^. Data on the onset and progression of periodontitis are still limited^2^. Generalized aggressive periodontitis (GAgP) is an inflammatory disease with an abnormal host response to specific bacterial plaque and this response is not consistent with the amount of bacteria. Aethiopathogenesis of aggressive and chronic periodontitis have been the focus of intense research.

The presence of periodontopathogens is required but not sufficient for initiation of periodontal disease^2^. The initiation and progression of periodontal disease depends on complex interactions between periodontopathic bacteria and the host immune system. Innate immune system cells which includes neutrophils, monocyte/macrophages are the first line of cellular immune response to infectious agents^3^,^4^. Therefore, the profile of the innate immune system cells involved in both aggressive and chronic periodontitis has been widely studied^5^,^6^,^7^,^8^,^9^,^10^,^11^,^12^. Immunohistochemical studies have reported that local inflammatory response is characterized by an intense recruitment of polymorphonuclear leukocytes and B cells and antibody producing plasma cells both within the periodontal tissues^13^. Compared to gingival tissue and peripheral blood samples from periodontally healthy patients, a depressed T helper/T suppressor ratio has been reported in periodontal diseases^14^. These findings have been interpreted as the possibility of altered local immune regulation in periodontitis^14^. Progression of T cell dominated gingival lesion to B cell dominated periodontitis lesion have been reported in chronic periodontitis (CP) by several researchers^15^,^16^,^17^,^18^. However, Pietruska et al. reported that in aggressive periodontitis T cells still dominate the periodontal lesions as in gingival lesions^19^.

Considerable number of reports have been accumulated since the 1980s that have aimed at looking at the immunological profiles of the periodontitis cases using different techniques such as immunohistochemistry and flow cytometry^20^,^21^,^22^,^23^,^24^. However, in such studies immune system was regarded as the “healing” factor. The aim of this study was to determine the possible destructive effects of the activated cytotoxic T lymphocytes (CD8+/CD28+) that might contribute to the development and progression of periodontitis by utilizing two color FACSCanto flow cytometry to determine the distribution of lymphocyte subpopulations. Reports from other studies have suggested that local blood obtained from tissue site differ in chemical composition from peripheral blood^25^. Therefore, we also wanted to compare the local blood obtained from damaged tissue site, which is much easier to obtain than performing immunohistochemistry, with peripheral blood in cell composition to determine whether any such difference exist. The locally obtained blood from the defective site and the peripheral blood were compared to determine whether there is a difference in the level of the activated cytotoxic T lymphocytes between local or peripheral blood due to tissue damage.

## Methods

### Selection of subjects and clinical assesment

Demographic variables of the study population are provided in Table 1. Otherwise healthy thirteen patients with GAgP and eleven with CP were included in the study according to the current classification of periodontal disease^1^. Subjects in the GCP group were >30 years of age and had a minimum of six teeth with at least one site each with PD and CAL > 5 mm, ≥30% of sites with PD and CAL > 5 mm, and presence of BOP. Subjects in the GAgP group ranged from 25 to 30 years of age and had at least six incisors and/or first molars with PD and CAL > 5 mm, a minimum of six teeth other than first molars and incisors with PD and CAL > 5 mm, and presence of BOP. Also, all GAgP patients had familial history (at least one family member presenting or with a history for periodontal disease). All study subjects were systemically healthy, non-smokers and did not have any history of allergy to local anesthetics. The patients had at least five teeth in each quadrant. Five systemically and periodontally healthy volunteers were served as the control group. All healthy controls had at least one gingival recession site with Miller I–II recession to cover with coronally advanced flap and/or subepithelial connective graft procedure. The subjects were ranged in age from 19 to 40 years. The study protocol was approved by the Ethical Committee of Istanbul University (Approval no: 2011/1993-861) and the methods were carried out in accordance with the guidelines of the Declaration of Helsinki. Before the study, informed consents were obtained from all subjects. Following clinical measurements were recorded; Plaque Index (PI, Silness-Löe)^26^, Probing Depth (PD), Clinical Attachment Level (CAL), Bleeding on Probing (BoP). Measurements were taken at six sites around all teeth excluding third molars using a periodontal probe (Williams, Hu-Friedy, Chicago, IL). All measurements were reported as mean value except for the defect site probing depth (D-PPD) and defect site clinical attachment level (D-CAL) which were measured at single sites. For the patients with periodontitis, the site with deepest PD in the upper jaw was selected for local blood sampling. Local blood from healthy subjects were collected during mucogingival surgery from periodontally healthy non-inflamed sites. Exclusion criteria for all subjects included: previous periodontal therapy, antibiotic therapy in the previous 6 months, any systemic condition, pregnancy or lactation.

### Clinical procedures

A periodontally hopeless, preferably single rooted tooth was selected based on the initial periodontal examination. Then local anesthesia was applied to the sampling site. A mucoperiosteal crevicular incision which started at the bottom of the periodontal pocket and directed to the bone margin was made and no vertical incisions were used. Only the buccal papilla was elevated to get a quick access to the base of the periodontal bone defect. Immediately after accessing the base of the defect, heparinized capillary tubes were placed in the deepest site of the defect and first bleeding was collected in these tubes. Papilla was then sutured using non-resorbable silk sutures. The blood was then transferred in to sterilized Eppendorf tubes containing 20 μl heparin. Whole blood was drawn from antecubital vein form all subjects prior to collection of blood from the defect site and the specimens collected in K3 EDTA coated tubes. The samples were immediately transported to laboratory for analysis by flow cytometry. Immediately after opening the flap for root coverage, first blood was collected and processed the same way as the periodontitis groups from healthy controls.

### Lab analyses

**Figure 1:**
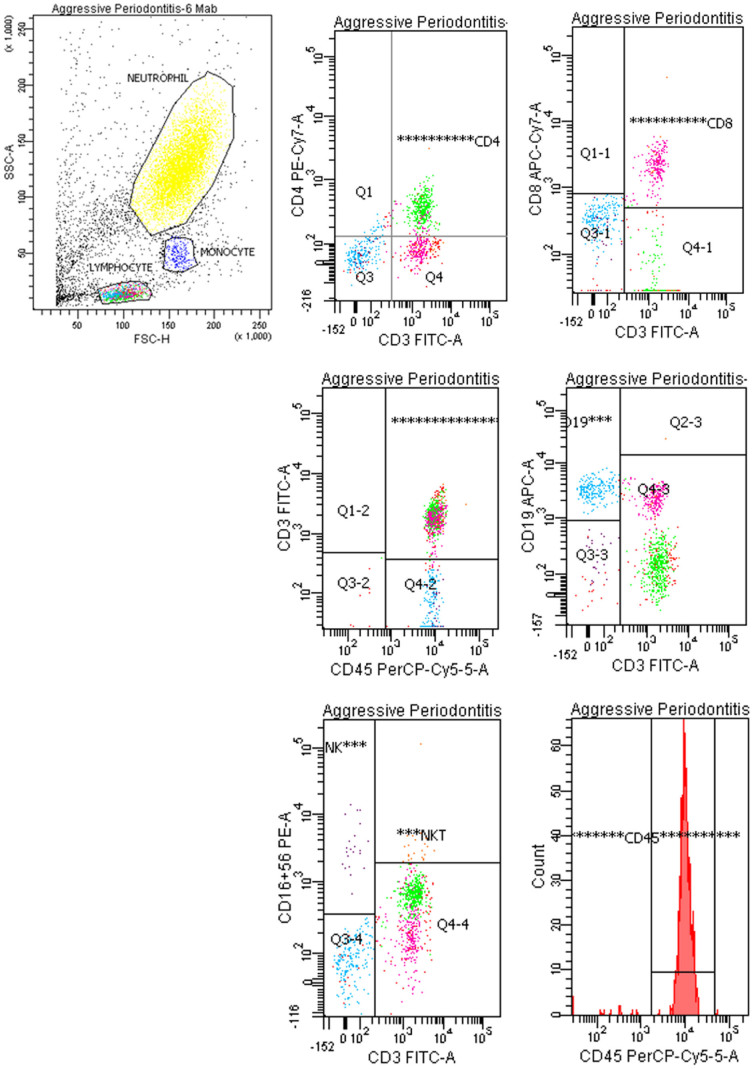
Flow cytometric analysis using six color Mab on FACSCanto with FACCSDiva software version 6.1.3. of a representative case.

**Figure 2:**
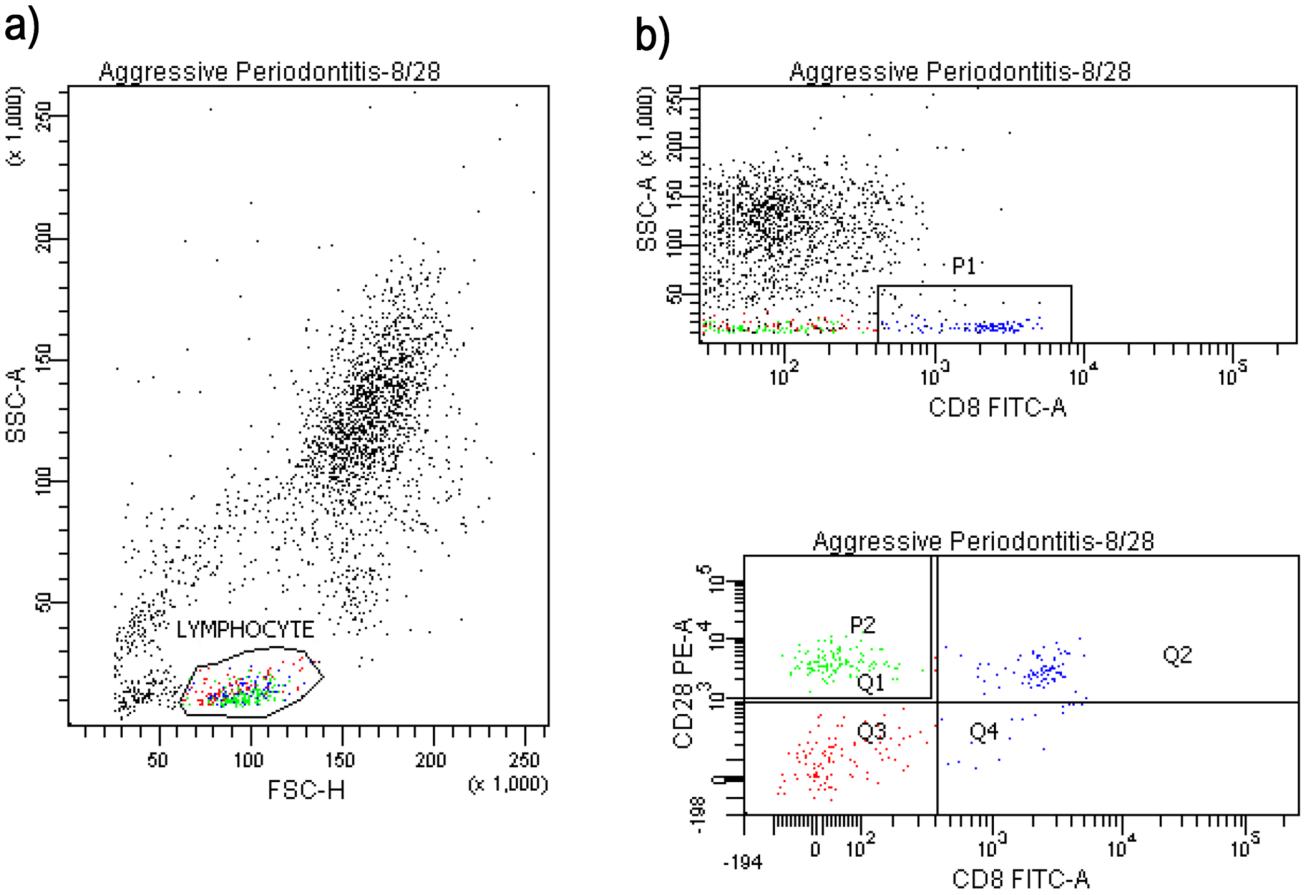
Flow Cytometric Analysis Using Double Mab CD8/CD28 Kit on FACSCanto with FACSDiva Software Version 6.1.3. of a representative case.

Peripheral whole blood and blood obtained from defect sites of the test groups and the control group were used for analysis. Lymphocytes were separated from blood by Ficoll histopaque-1077 density gradient (Sigma, St. Loui, MO). The white cell interface (buffy coat) was transferred in to collection tubes and was washed two times in 1 × PBS solution by centrifugation. Following two washes, for each sample, 1 × 10^6^ cells/ml were stained with fluorochrome-conjugated monoclonal antibodies for specific receptors: CD45 (common leucocyte marker), CD4 (T helper cell marker), CD3 (lymphocyte and NKT marker), CD19 (B lymphocyte marker), CD8/CD28 (cytotoxic T cell marker), CD16/CD56 (NK and NKT markers) according to manufacturer's instructions (6 and 2 color combination kits, BD Multitest 6 color TBNK, Catalog no: 644611, BD simultest CD8/CD28, Catalog no: 340031, BD and Pharmingen-San Diego, CA). For staining, 20 μl monoclonal antibody was added per 100 μl whole blood sample, incubated for 20 min. at 37°C in the dark and mixed by vortexing, and the samples were incubated in 2 ml lysis solution for another 10 min. in the dark followed by centrifugation at 1310 rpm for 5 min. at +4°C supernatant was removed and pellet vortexed. Then the samples were washed twice: 2 ml facs flow was added, centrifuged at 1310 rpm for 5 min., supernatant was removed, remaining pellet was vortexed. Finally 500 μl facs flow was added and vortexed and analysed with Facs. All samples were loaded onto FACSCanto Flow Cytometer (BD Biosciences, San Jose, CA) for analysis with BD FACSDiva software (BD Biosciences, San Jose, CA) for manual immunophenotyping assays prepared using the lyse/wash method as described above. At least 10.000 cells were counted in appropriate lymphocytes gate for each tube's sample in flow cytometry by using FACSDiva software program and then the percentage values of all markers were determined using appropriate analyzing programme. The analyzed receptors and related cells are as follows: CD3^+^/CD16^+^/CD56^+^ cells were regarded as NKT cells, CD3^−^/CD16^+^/CD56^+^ cells were regarded as NK cells and CD8^+^/CD28^+^ cells were regarded as activated cytotoxic T cells whereas CD8^+^/CD28^−^ cells were considered as the resting cytotoxic T cells. Figures 1 and 2 summarizes the flow cytometry protocol for lymphocyte detection and the gating strategy used in the study.

### Statistical Analyses

Prior to initiation of the study, power and sample size was calculated. Type-one error was assumed at 0.05, type-two error was assumed at 0.2 and power was assumed at 0.80. The minimum sample size was calculated to consist of 10 subjects for each group. Differences among the study groups (clinical, demographic and biochemical categories) were assessed by ANOVA for the parametric data and Kruskal-Wallis for the non-parametric data. Bonferroni corrected Kruskal-Wallis Multiple Comparison Z-Value Test was used to determine if there were “significant” differences among the population medians. Differences within groups were analyzed by Wilcoxon test for non-parametric data and independent t test for parametric data. Statistical significance was accepted as P < 0.05. All results were evaluated at 95% confidence interval. Statistical software was used for analysis Pass 2008/NCSS 2007, NCSS, Kaysville, UT.

## Results

Demographic profile and periodontal variables are shown in Table 1. There was no difference in periodontal findings between GAgP and CP groups based on whole mouth averages of periodontal probing depth, clinical attachment loss and PPD and CAL measurements of the defect sites. There was no difference in both periodontitis groups in terms of periodontitis activity as assessed by BoP scores. Plaque index scores were significantly higher in CP group than GAgP and healthy controls. However, CP group comprised of significantly elderly patients compared to GAgP and HC groups.

**Figure 3:**
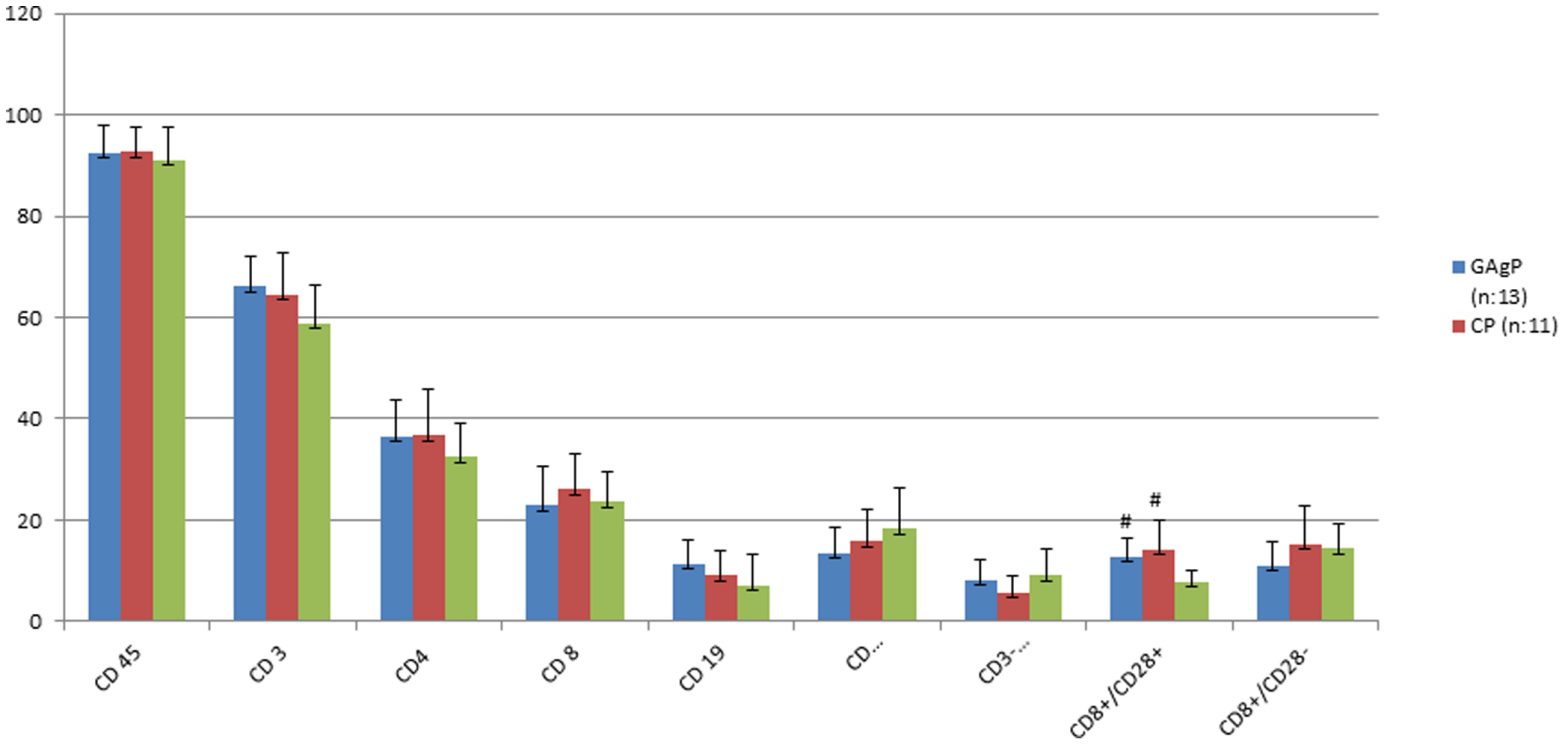
Phenotypic lymphocyte subset analysis of GAgP, CP and HC groups in blood from the defect site (% proportion of lymphocytes positive for the given monoclonal antibodies)

**Figure 4:**
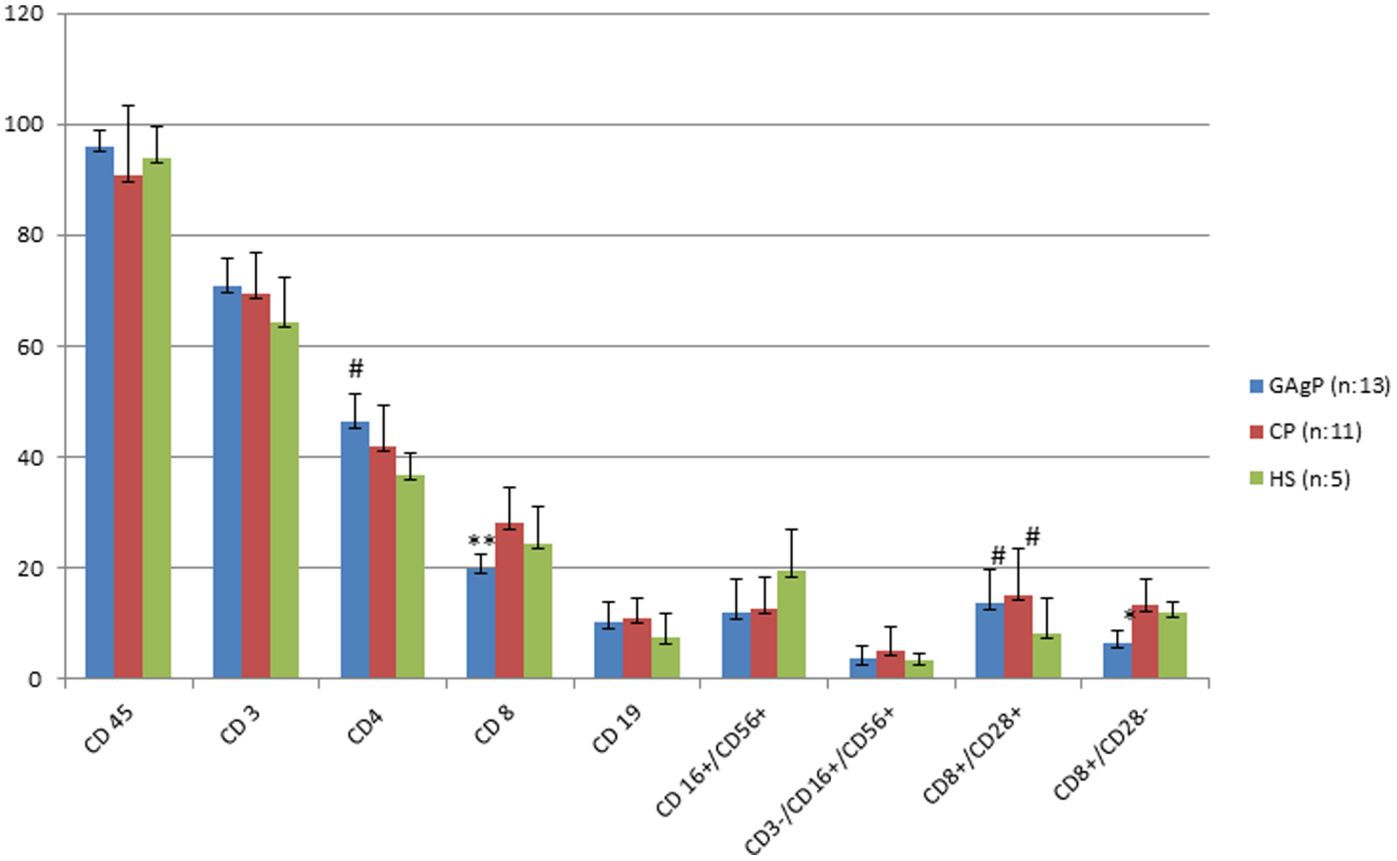
Comparison of % proportion of lymphocytes positive for the various monoclonal antibodies in peripheral blood among study groups

Phenotypic lymphocyte subset analysis of GAgP, CP and HC groups in blood from defect site is shown in Table 2 and Figure 3. There was no statistically significant difference in any lymphocyte profiles except for CD8^+^/CD28*^+^* cells among GAgP, CP and HC groups which was significantly higher in both periodontitis groups compared to healthy controls (p < 0.05). Phenotypic lymphocyte subset analysis of GAgP, CP and HC groups in systemic blood is shown in Table 3 and Figure 4. Similar to results obtained from defect site blood, CD8^+^/CD28*^+^* was found to be significantly higher in both periodontitis group than healthy subjects in systemic blood (p < 0.05). However, systemic levels of CD8^+^/CD28^−^ were significantly lower in GAgP patients compared to CP and healthy controls (p = 0,009; ANOVA, z = 2,931; KW).

The data was analyzed to detect possible differences between systemic and defect site circulation levels of each lymphocyte profile within either study groups. Comparison of local and systemic levels of each molecule within groups is demonstrated in Table 4. More pronounced and statistically significant changes were visible in GAgP group. CD45, CD3 and CD4 molecule levels were significantly higher (p <0.05) in systemic circulation of GAgP patients compared to defect site circulation levels of the same group. In the contrary, CD3^+^/CD16^+^/CD56^+^, and CD8^+^/CD28^−^ molecules were significantly higher in defect site blood compared to systemic blood levels of GAgP group. Comparison of defect site and systemic levels of study molecules in whole periodontitis population (GAgP plus CP as a single periodontitis group) is also demonstrated in Table 4. CD3 and CD4 were significantly higher in systemic circulation (p < 0.05) contrary to CD3^+^/CD16^+^/CD56^+^ cells that were found in significantly higher levels in local circulation (p < 0.05). However no significant difference was observed in levels of CD8^+^/CD28^+^ cells in either blood samples (p > 0.05).

## Discussion

In this study we aimed to examine the possible involvement of cytotoxic T cells and its activation status in the pathogenesis of generalized aggressive periodontitis and chronic periodontitis. Our results indicated that there is a significant increase in activated cytotoxic T cells in both GAgP and CP patients compared to HC. Several studies examined the CD8^+^ cytotoxic T cells but did not identify whether these cells were activated^13^,^22^,^27^. To the best of our knowledge none of the previous studies have mentioned about the involvement of activated cytotoxic T cells as a contributing factor to the pathogenesis of periodontitis.

In response to an infection, innate immune system components act as the first line of defense followed by a more specific adaptive immune response that include humoral (B cell mediated) and cellular (T cell mediated) arms to control the infection and disease progression. The level of activated cytotoxic T cells (Tc) are controlled by regulatory T (Treg) cells after the Tc cells have completed their mission. Uncontrolled activation of Tc cells lead to tissue damage^28^. When the host-bacterial interaction that causes to tissue destruction in periodontal disease is considered, the activation of Tc cells might have a role on the progression of periodontal disease. Therefore, our null hypothesis was that the activated Tc in GAgP and CP might be higher than HC. Early studies utilizing immunohistochemistry and flow cytometry have shown increased level of innate immune cells and activated humoral immune response in the defective tissue and peripheral blood^13^,^20^,^21^,^22^,^23^,^24^,^27^,^29^,^30^. It is possible that during the course of an uncontrolled bacterial infection, activated cytotoxic T cells cause tissue damage.

The destruction of the periodontal attachment during periodontitis is primarily caused by the mechanisms leading to the damage of protein components of connective tissue. Although a great number of reports are published on etiopathogenesis of periodontal disease, the factors contributing to severe disease progression have not been fully understood. However, since the publication of the first reports, there have been changes in laboratory techniques and in our understanding of how immune system functions in the regulation of responsiveness to periodontopathogens in the peripheral blood.

Concerning the other lymphocyte subgroups examined in the current study, we have found similar CD3, CD19, CD45 and CD3−/16+/56+ cell levels of the GAgP and CP groups compared to HC as consistent with the results of Buduneli et al.^23^, and Zafiropoulos et al^30^. Emingil et al. also found similar CD4+ and CD8+ levels between the same periodontitis groups and healthy controls^24^. However Afar et al. reported higher levels of T and B lymphocytes in periodontitis patients compared to healthy controls in contrast to our results^31^.

We did not find any difference in local blood profile between both periodontitis groups. However, increased Tc cells in peripheral blood in both periodontitis groups indicates that T cell levels in peripheral blood is not dependent of the type of periodontitis. There was no increase in activated Tc cells locally or peripherally in healthy controls which supports the phenomenon that periodontal disease has an impact on systemic immune response.

Progression of gingivitis into severe forms of periodontitis indicates that other factors must be involved in disease progression despite expected innate and humoral immune response which could be the increase level of uncontrolled activated Tc cells.

### Limitations of the Study

The study has several limitations: Some disciplines use local blood sampling rather than systemic sampling to study chemical composition of blood^25^. In light of this study, we wanted to see if the blood taken directly from the damaged tissue would provide us any information with regards to cell population levels compared to blood taken from the systemic circulation as a novel approach in periodontal disease so that this method could be used as an alternative to immunohistochemistry which is a more cumbersome method. Therefore we did not perform immunohistochemistry.

We also know that it is important to examine the level of Treg cells to determine whether the increased level of activated Tc cells is due to low level of Tregs. In order to better understand the effect of cellular immune dysfunction on progression of periodontitis, higher number of cases along with Treg level determination needs to be carried out.

In addition, we primarily classified the cells as activated or non-activated CTLs based on the expression of CD28. We are aware that additional markers like CD69, CD40L or more specific/functional level markers like IFNg expression on these cell subsets should also be the subject of further studies to confirm our hypothesis. Besides, the expression of other molecular signatures like Fox P3, IL-17A, RORgc and other inflammatory signatures on different cell population might be interesting to see the difference by techniques like QRT-PCR and support this hypothesis. However, currently we did not have the means to be able to perform the studies.

Although study has several major limitations, the results of this study may diverge the attention of the researchers with more sophisticated immunological research tools to study the contribution of immune system cells on periodontal disease progression.

Within the limitations of our study, we can conclude that activated Tc cells might have a potential role in the destruction of periodontal tissue. Intervention studies would provide more insight about the net effect of these cells in the etiopathogenesis of periodontal disease. The results of these studies might allow changes in treatment strategies of the periodontitis cases with probable use of immune modulators.

## Author Contributions

E.C. and M.C. wrote the main manuscript. B.K. prepared the tables 2–4. S.B. and E.F. designed the study. H.I. made the data analyses. S.C. revised the manuscript critically for important intellectual content. All authors reviewed the manuscript.

## Figures and Tables

**Table 1:** Demographic profile and periodontal variables of the study population.

	GAgP (n:13)	CP (n:11)	HC (n:5)	p-value (ANOVA)
**Age**	26.31 ± 5.77**	34.82 ± 3.92	25.2 ± 4.33**	0
**Gender (%f)**	61%	54%	60%	NS
**PI**	0.97 ± 0.56**	1.67 ± 0.88	0.87 ± 0.45**	0,03
**BoP**	52.45 ± 22.87#	46.61 ± 39.86#	5 ± 3.16	0.01
**PPD**	2.69 ± 0.56#	2.72 ± 0.36#	1.451 ± 0.11	0
**CAL**	3.11 ± 0.64#	3.36 ± 0.72#	1.341 ± 0.07	0
**D-PD**	6.38 ± 1.38#	5.27 ± 1.10#	2 ± 0.0	0
**D-CAL**	6.77 ± 1.73#	5.64 ± 1.43#	2 ± 0.00	0

**GAgP**: Generalized aggressive periodontitis.

**CP**: Chronic periodontitis.

**HC**: Healthy controls.

**PI**: Plaque Index.

**BoP**: Bleeding on Probing as percentage.

**PPD**: Periodontal Probing Depth.

**CAL**: Clinical Attachment Level.

**D-PPD**: Defect site periodontal probing depth.

**D-CAL**: Defect site clinical attachment level.

#compared to healthy subjects.

**compared to the CP group.

**Table 2:** Phenotypic lymphocyte subset analysis of GAgP, CP and HC groups in blood from the defect site (% proportion of lymphocytes positive for the given monoclonal antibodies)

	GAgP (n:13)	CP (n:11)	HC (n:5)
**CD 45**	92.54 ± 5.50	92.64 ± 4.82	91.2 ± 6.22
**CD 3**	66.08 ± 6.02	64.55 ± 8.28	58.8 ± 7.53
**CD4**	36.62 ± 7.05	36.73 ± 8.97	32.4 ± 6.58
**CD8**	22.92 ± 7.65	26.09 ± 6.93	23.6 ± 5.77
**CD 19**	11.23 ± 4.96	9.09 ± 4.82	7.2 ± 6.18
**CD16^+^/CD56^+^**	13.54 ± 4.97	15.73 ± 6.24	18.2 ± 8.04
**CD3^−^/CD16^+^/CD56^+^**	8.15 ± 4.05	5.73 ± 3.10	9 ± 5.38
**CD8^+^/CD28^+^**	*12.77 ± 3.65*#	*14.25 ± 5.65*#	*7.8 ± 2.38*
**CD8^+^/CD28^−^**	11.77 ± 4.54	15.29 ± 7.54	14.40 ± 5.03

**GAgP**: Generalized aggressive periodontitis.

**CP**: Chronic periodontitis.

**HC**: Healthy controls.

#: Significantly higher levels in percent CD8^+/^CD28^+^ cells compared to healthy control.

Bonferroni corrected Kruskal-Wallis Multiple Comparison Z test was performed.

CD45: Common leucocyte marker,

CD4: T helper cell marker,

CD3: lymphocyte and NKT marker,

CD19: B lymphocyte marker,

CD8/CD28: Cytotoxic T cell marker,

CD16/CD56: NK and NKT markers,

CD3+/CD16+/CD56+: NKT cells,

CD3−/CD16+/CD56+: NK cells,

CD8+/CD28+: Activated cytotoxic T cells,

CD8+/CD28−: Resting cytotoxic T cells.

**Table 3:** Comparison of % proportion of lymphocytes positive for the various monoclonal antibodies in peripheral blood among study groups

	GAgP (n:13)	CP (n:11)	HC (n:5)
**CD 45**	96.08 ± 2.90	90.64 ± 12.82	94 ± 5.43
**CD 3**	70.77 ± 5.01	69.55 ± 7.20	64.4 ± 7.89
**CD4**	46.23 ± 5.31#	42.09 ± 7.14	36.8 ± 3.76
**CD 8**	20 ± 2.44**	28 ± 6.64	24.4 ± 6.76
**CD 19**	11.23 ± 4.96	9.09 ± 4.82	7.2 ± 6.18
**CD3−/CD16^+^/CD56^+^**	11.92 ± 6.04	12.73 ± 5.51	19.4 ± 7.50
**CD3^+^/CD16^+^/CD56^+^**	3.62 ± 2.32	5.27 ± 4.07	3.4 ± 1.34
**CD8^+^/CD28^+^**	13.54 ± 2.25#	15.09 ± 4.76#	8.2 ± 2.04
**CD8^+^/CD28^−^**	6.50 ± 1.88*	13.29 ± 7.34	12 ± 3.93

**GAgP**: Generalized aggressive periodontitis.

**CP**: Chronic periodontitis.

**HC**: Healthy controls.

#: significantly higher levels in CD4 (in GAgP group) and CD8^+^/CD28^+^ levels (in GAgP and CP group) compared to healthy subjects.

*significantly lower levels of CD8^+^/CD28^−^ cells in GAgP group compared to HC and CP groups.

**significantly lower CD 8 levels in GAgP group compared to the CP group.

Bonferroni corrected Kruskal-Wallis Multiple Comparison Z test was performed.

**Table 4:** Local and Systemic Cell Count Comparison within GAgP, CP, GAgP+ CP sand Healthy Controls (% proportion of lymphocytes positive for the various monoclonal antibodies)

Molecules	Groups	Median	Minimum	Maximum	p-value
**CD45 systemic/defect**	GAgP	96/94	88/80	99/99	0.00*
	CP	96/94	53/82	97/98	0.96
	HC	96/90	85/82	98/97	0.22
	GAgP+CP	96/94	53/80	99/99	0.07
**CD3 systemic/defect**	GAgP	71/66	62/62	78/76	0.02*
	CP	69/65	61/53	81/78	0.04
	HC	62/61	56/46	77/66	0.13
	GAgP+CP	71/65	61/53	81/78	0.00*
**CD4 systemic/defect**	GAgP	46/38	39/39	57/50	0.00*
	CP	44/37	29/22	52/20	0.02
	HC	37/36	31/23	41/38	0.06
	GAgP+CP	44,5/38	29/22	57/50	0*
**CD8 systemic/defect**	GAgP	20/23	17/8	25/41	0.08
	CP	29/27	17/13	40/38	0.05
	HC	21/21	19/18	34/32	0.89
	GAgP+CP	21/24	17/8	40/41	0.72
**CD19 systemic/defect**	GAgP	11/11	4/30	16/21	0.23
	CP	12/7	6/1	16/6	0.16
	HC	8/5	2/11	12/7	0.78
	GAgP+CP	11,5/10,5	4/1	16/21	0.92
**CD3^−^/CD16^+^/CD56^+^ systemicdefect**	GAgP	11/14	2/6	25/23	0.23
	CP	13/14	5/9	23/29	0.10
	HC	20/14	9/11	29/30	0.68
	GAgP+CP	1/12	2/6	25/29	0.05
**CD3^−^/CD16^+^/CD56^+^ systemic/defect**	GAgP	3/8	1/2	7/16	**0.00**‡
	CP	4/5	1/1	16/10	0.48
	HC	4/8	6/5	5/18	**0.04**‡
	GAgP+CP	4/7	1/1	16/16	**0.00**‡
**CD8^+^/CD28^+^ systemic/defect**	GAgP	14/11	10/8	17/20	0.38
	CP	13/13,5	11/6	27/23	0.67
	HC	9/8	6/5	5/18	0.04
	GAgP+CP	13,5/11	10/6	27/23	0.33
**CD8^+^/CD28^−^Systemic/defect**	GAgP	6,5/11,5	4/6	9/21	**0.00**‡
	CP	12/14	7/8	29/31	0.14
	HC	10/14	9/8	18/22	0.09
	GAgP+CP	11/8	6/4	23/29	0.00*

**GAgP**: Generalized aggressive periodontitis.

**CP**: Chronic periodontitis.

**HC**: Healthy controls.

*: significantly lower molecule levels in blood obtained from defect site compared to systemic blood (p <0.05).

‡: significantly higher molecule levels in blood obtained from defect site compared to systemic blood (p <0.05).
